# Autosomal recessive osteopetrosis: mechanisms and treatments

**DOI:** 10.1242/dmm.048940

**Published:** 2021-05-10

**Authors:** Sara Penna, Anna Villa, Valentina Capo

**Affiliations:** 1 San Raffaele Telethon Institute for Gene Therapy (SR-Tiget), IRCCS San Raffaele Scientific Institute, Milan 20132, Italy; 2 Translational and Molecular Medicine (DIMET), University of Milano-Bicocca, Monza 20900, Italy; 3 Institute of Genetic and Biomedical Research, Milan Unit, National Research Council, Milan 20090, Italy

**Keywords:** Osteopetrosis, Bone disease, Osteoclast, Hematopoietic stem cell transplantation, Gene therapy

## Abstract

Autosomal recessive osteopetrosis (ARO) is a severe inherited bone disease characterized by defective osteoclast resorption or differentiation. Clinical manifestations include dense and brittle bones, anemia and progressive nerve compression, which hamper the quality of patients' lives and cause death in the first 10 years of age. This Review describes the pathogenesis of ARO and highlights the strengths and weaknesses of the current standard of care, namely hematopoietic stem cell transplantation (HSCT). Despite an improvement in the overall survival and outcomes of HSCT, transplant-related morbidity and the pre-existence of neurological symptoms significantly limit the success of HSCT, while the availability of human leukocyte antigen (HLA)-matched donors still remains an open issue. Novel therapeutic approaches are needed for ARO patients, especially for those that cannot benefit from HSCT. Here, we review preclinical and proof-of-concept studies, such as gene therapy, systematic administration of deficient protein, *in utero* HSCT and gene editing.

## Abstract (precis)

**Summary:** Autosomal recessive osteopetrosis is a heterogeneous and rare bone disease for which effective treatments are still lacking for many patients. Here, we review the literature on clinical, preclinical and proof-of-concept studies.

## Introduction

Osteopetrosis, or marble bone disease, was first described in 1904 by the German radiologist Albers Schönberg as a heritable disorder characterized by increased bone density, typically described as ‘bone within bone’ appearance (Stark and Savarirayan, 2009). The impaired equilibrium of bone formation and remodeling leads to structural brittleness, predisposition to fractures, skeletal deformities and dental abnormalities (Sobacchi et al., 2013). The disease is caused by defective osteoclast differentiation or function, but is clinically and genetically heterogeneous, ranging in severity from benign to lethal in early childhood (Penna et al., 2019). Based on the pattern of inheritance, osteopetroses have been categorized into autosomal recessive osteopetrosis (ARO), also known as infantile malignant osteopetrosis, and autosomal dominant osteopetrosis, an adult-onset more benign form (Palagano et al., 2018). In this Review, we will focus on ARO and its pathogenesis, as it represents the most severe osteopetrosis with a strong unmet clinical need. We will discuss the standard of care and the ongoing clinical trials, as well as innovative treatments that are currently being developed and can be applied to ARO.

## Pathogenesis of ARO

ARO has an incidence of 1:250,000 live births and is fatal within the first decade of life if untreated (Sobacchi et al., 2013). Patients suffer severe symptoms, including growth retardation, skull abnormalities (macrocephaly, frontal bossing, choanal stenosis), hydrocephalus, hypocalcemia (owing to the defective calcium mobilization activity of osteoclasts) and abnormal tooth eruption (Wu et al., 2017). The abnormal bone density causes an expansion of skeletal tissue into marrow cavities at the expense of the bone marrow niche, leading to severe anemia, bleeding, frequent infections and hepatosplenomegaly due to increased extramedullary hematopoiesis. The increased susceptibility to infections leads to the development of dental caries and facial osteomyelitis, especially after dental surgery (Mikami et al., 2016). The limited bone marrow niches cause the circulation of high numbers of hematopoietic stem and progenitor cells (HSPCs; positive for CD34 marker) in the peripheral blood, especially in young patients with severe forms (Capo et al., 2021; Steward et al., 2005). However, the mechanisms underlying the spontaneous CD34^+^ mobilization from the bone marrow niche of ARO patients still remain poorly characterized. Moreover, the encroachment of cranial nerve foramina leads to blindness, deafness and nerve palsies (Wu et al., 2017).

**Fig. 1.**
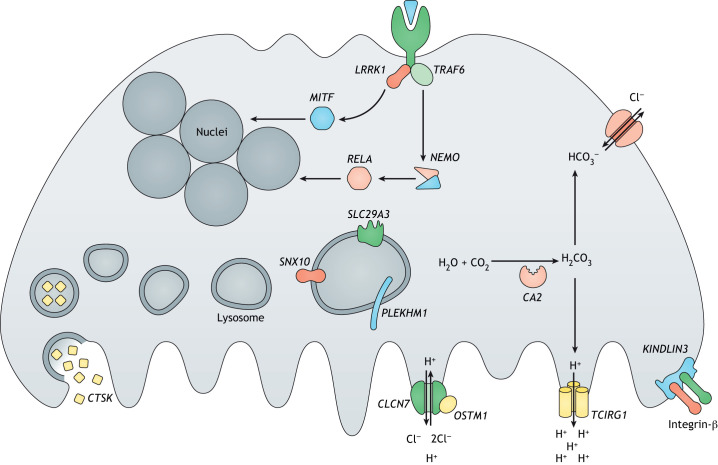
**Schematic representation of genes involved in osteoclast-rich osteopetrosis.** The figure shows genes involved in the bone resorptive activity of osteoclasts with different functions, including acidification of resorption lacunae and pH regulation (*TCIRG1*, *CLCN7*, *OSTM1* and *CA2*), vesicular trafficking and sorting of protein complexes to the membrane (*SNX10* and *PLEKHM1*), lysosomal nucleoside trafficking (*SLC29A3*), cytoskeletal rearrangement for ruffled border formation (*KINDLIN3*, integrin-β and *LRRK1*) and lysosomal proteolytic cleavage for bone remodeling and resorption (*CTSK*). Moreover, genes that are involved in different signal transduction pathways and essential for osteoclast function (*MITF*, *TRAF6*, *RELA* and *NEMO*) have been reported. Figure originally created using Servier Medical Art (http://smart.servier.com/), licensed under a CC-BY 3.0 license, and re-drawn according to journal style.

**Fig. 2.**
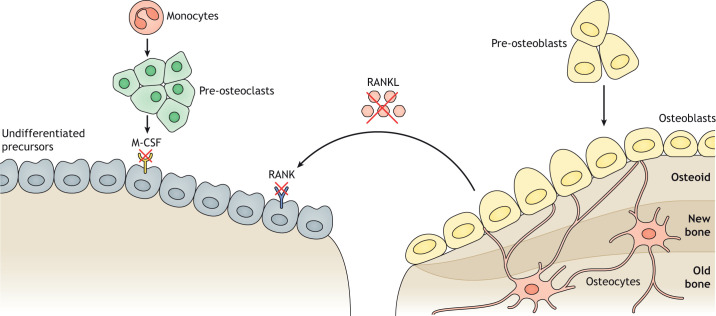
**Representation of proteins involved in osteoclast-poor osteopetrosis.** Impaired crosstalk between osteoclasts and osteoblasts gives rise to deficient bone remodeling. In osteoclast-poor osteopetrosis, the osteoclast differentiation pathway is impaired due to mutations in the *TNFSF11* and *TNFRSF11A* genes, encoding RANKL and its receptor RANK, respectively, or in the *CSF1R* gene, encoding M-CSF. As a consequence, osteoclast precursors are not able to fuse and to differentiate into multinucleated resorbing osteoclasts. Mutated genes are indicated by a red cross. Figure originally created using Servier Medical Art (http://smart.servier.com/), licensed under a CC-BY 3.0 license, and re-drawn according to journal style.

ARO disease originates from mutations in different genes that are involved in osteoclast functions (osteoclast-rich osteopetrosis) (Fig. 1) or differentiation (osteoclast-poor osteopetrosis) (Fig. 2) (Penna et al., 2019).

### Osteoclast-rich osteopetroses

Among osteoclast-rich forms (Fig. 1), more than 50% of ARO cases are caused by mutations in the T cell immune regulator 1 (*TCIRG1*) gene, which encodes the V-type proton ATPase 116 kDa subunit a3 (OC116). It acidifies the bone resorption lacunae (see Glossary, Box 1), favoring the dissolution of hydroxyapatite crystals, which form the bone mineral fraction, and the degradation of the organic bone matrix (Frattini et al., 2000). Moreover, OC116 plays a role in the interaction between the actin cytoskeleton and microtubules, which is essential for osteoclast ruffled border (Box 1) formation (Matsumoto et al., 2018). The *TCIRG1* gene is expressed in most tissues, in particular by osteoclasts and gastric parietal cells on the apical membrane. In the stomach, the V-ATPase proton pump maintains the low pH essential for dietary Ca^2+^ absorption; thus, ARO patients also present rickets or osteomalacia due to defective calcium uptake (Schinke et al., 2009).

**Box 1. Glossary**

**Autologous back-up transplantation:** before conditioning, autologous hematopoietic stem cells (HSCs) are harvested and stored as back-up to be potentially reinfused to the patient in case of graft failure or severe graft versus host disease (GvHD). Autologous transplant does not cure the disease but allows rescue of the patient’s bone marrow as a bridge therapy to a second allogeneic transplant.

**Circulating CD34^+^ cells:** peripheral blood CD34^+^ cells are a heterogeneous population of hematopoietic stem/progenitor cells (HSPCs) that circulate outside the bone marrow niche. In physiological conditions, low numbers of CD34^+^ cells are found in peripheral blood, while the lack of bone marrow niches in osteopetrotic bones induces increased egress from bone marrow.

**C-terminal telopeptide of type I collagen (CTX-I):** type I collagen is the most abundant component of the organic matrix of bone. During the bone remodeling process, type I collagen is degraded, and small peptide fragments, such as CTX-I, are excreted into the bloodstream. Serum levels of these degradation products can be quantified as a marker of bone resorption.

**Disease-free survival**: the term identifies the time after HSC transplantation (HSCT), during which the patients survive with signs of osteoclast function (hypercalcemia, improvement by radiographs, improvement of hepatosplenomegaly). However, irreversible nerve damage or bone deformities occurring before HSCT are still present.

**Donor chimerism:** the percentage of donor cells that have durably engrafted in the recipient. The chimerism is complete if 100% of bone marrow and blood cells are of donor origin, whereas it is mixed or partial if recipient cells persist.

**Exchange apheresis:** the apheresis machine allows the separation and collection of CD34^+^ cells from the other blood components by centrifugation, returning all other blood components to the patient. The procedure allows the processing of a large volume of blood to collect HSCs, limiting side effects of multiple blood drawings.

**Haploidentical HSCT:** an HSCT in which the source of HSCs derives from a human leukocyte antigen (HLA) half-matched donor, usually a parent or sibling of the patient.

**Podosome:** conical, actin-rich structure located on the outer surface of the plasma membrane. Podosomes play a crucial role in the bone remodeling process, connecting osteoclast precursors to the bone surface. When the bone resorption process starts, they become a single actin ring, delimiting the sealing zone of mature osteoclasts on the bone resorption site.

**Reduced intensity conditioning (RIC):** a conditioning regimen that does not completely abolish host bone marrow, with decreased toxic effect on the bone marrow niche compared to the standard myeloablative approach.

**Resorption lacunae:** also called Howship's lacunae, are tiny depressions, pits or irregular grooves in bone that is being resorbed by osteoclasts.

**Ruffled border:** in close proximity to the bone, at the resorption lacunae, the osteoclasts’ membrane is characterized by finger-like processes composing the ruffle border. This extensively folded border increases the cell surface able to host the components of the bone resorption machinery of osteoclasts.

**Sealing zone:** specialized cell–matrix adhesion structure formed at the junction between mature osteoclast and bone, that assembles during bone resorption. It is a dynamic actin-rich structure that defines the osteoclast resorption area of the bone.

**Sleeping Beauty transposon**: a system that allows the transposition of a synthetic DNA sequence in the genome, through a cut-and-paste process. The integration of transposon into host DNA guarantees long-term transgene expression in transgenic cells and organisms.

**T cell-depleted haploidentical HSCT:** an allogeneic transplantation in which CD34^+^ cells are purified before the infusion in order to deplete T cells from the graft. In the non-identical setting, the use of T cell-depleted transplants prevents GvHD.

**UM171:** a pyrimidoindole derivate that supports the expansion of HSPCs while maintaining their stemness features and engraftment capacity.

Milder forms of osteopetrosis can be diagnosed later in life, accounting for a small number of patients with slower disease progression (Howaldt et al., 2019). In particular, recent studies reported novel single-nucleotide changes in the *TCIRG1* gene that cause aberrant splicing and exon skipping, thereby reducing the splicing efficiency without completely abrogating the production of the normal transcript (Palagano et al., 2015; Zirngibl et al., 2019).

The second most frequent form of osteopetrosis (17% of ARO cases) is caused by mutations in the chloride voltage-gated channel 7 (*CLCN7*) gene. The *CLCN7* gene encodes H(+)/Cl(−) exchange transporter 7 (ClC-7), a 2Cl^−^/H^+^ antiporter that is expressed at the osteoclast ruffled border and on the membrane of late endosomes and lysosomes, and that is regulated by a voltage-gating mechanism (Leisle et al., 2011). Because this channel is associated with the V-ATPase, it plays a fundamental role in maintaining an acid pH at resorption lacunae. It is also involved in vesicle trafficking in early and recycling endosomes, regulating the luminal Cl^−^ concentration in the kidney and central nervous system (CNS) (Novarino et al., 2010).

Depending on the type of mutation, patients present with mild or severe forms characterized by a wide variety of clinical manifestations (Kornak et al., 2001; Palagano et al., 2018; Pangrazio et al., 2010). In particular, symptom presentation varies from ‘classical’ infantile osteopetrosis with or without progressive neurodegeneration (displaying pathological electroencephalography), to intermediate autosomal osteopetrosis (IAO) and even to benign autosomal dominant osteopetrosis. In terms of genetic characterization, biallelic mutations of the *CLCN7* gene are reported to cause severe forms of osteopetrosis, showing bone damage, hematological failure and primary neurodegeneration, with symptoms resembling lysosomal storage defects (Kornak et al., 2001; Sobacchi et al., 2007). Neurodegeneration is probably due to the defective localization of ClC-7 in lysosomal membranes, because patients bearing mutations that maintain correct membrane targeting do not present CNS involvement (Di Zanni et al., 2021). Additionally, *CLCN7* mutations could account for the milder IAO, when patients suffer from mild symptoms and reach adulthood. Conversely, single-allele *CLCN7* mutations lead to autosomal-dominant osteopetrosis and are associated with milder symptoms and later onset (Li et al., 2019a; Sobacchi et al., 2013).

Less frequent osteopetroses (5% of ARO cases) present mutations in the osteoclastogenesis-associated transmembrane protein 1 (*OSTM1*) gene, encoding a highly glycosylated protein at its N-terminus able to stabilize ClC-7 and complement its activity at the ruffled border (Chalhoub et al., 2003; Leisle et al., 2011). A similar frequency of ARO cases is reported for mutations in *SNX10*, encoding the protein sortin nexin-10, targeting OC116 at the ruffled border (Pangrazio et al., 2013). SNX10 deficiency causes abnormal osteoclastogenesis, leading to relatively variable clinical manifestations, mainly affecting bone tissue (Aker et al., 2012).

Extremely rare forms of osteoclast-rich osteopetrosis are caused by defects in the carbonic anhydrase 2 (*CA2*) (Sly et al., 1983), pleckstrin homology and RUN domain-containing M1 (*PLEKHM1*) (Van Wesenbeeck et al., 2007), leucine-rich repeat kinase 1 (*LRRK1*) and melanocyte-inducing transcription factor (*MITF*) genes (Howaldt et al., 2020; Iida et al., 2016; Lu et al., 2014). The pathogenesis of these forms is poorly characterized due to the small number of identified patients. Other mutations have been reported to cause osteopetrosis as secondary disease among other clinical manifestations. In particular, patients with leucocyte adhesion deficiency III can display high bone mass due to mutations disrupting fermitin family homolog 3 [Kindlin-3 protein; encoded by the FERM domain-containing kindlin 3 (*FERMT3*) gene] and the integrin-β pathway, which cause the abrogation of podosome (Box 1) and sealing zone (Box 1) formation, which are required for osteoclast bone resorption (Malinin et al., 2009; Schmidt et al., 2011). Additionally, osteopetrosis may be secondary to the bone pathology arising from cathepsin K (*CTSK*) mutations, which is termed pycnodysostosis in humans (Gelb et al., 1996).

### Osteoclast-poor osteopetroses

Mutations affecting osteoclast differentiation are responsible for the osteoclast-poor forms of osteopetrosis (Fig. 2). In particular, loss-of-function mutations impairing the expression of the receptor activator of nuclear factor kappa-Β ligand (RANKL) cytokine or its receptor, RANK, have been reported to cause osteopetrosis in 2% and 4.5% of ARO cases, respectively. The TNF superfamily member 11 (*TNFSF11*) gene encodes RANKL, the master osteoclastogenic cytokine produced mainly by osteoblasts and stromal cells in bone (Lacey et al., 1998), whereas TNF receptor superfamily member 11a (*TNFRSF11A*) encodes RANK, a receptor mainly expressed by osteoclast precursors and mature osteoclasts (Nakagawa et al., 1998). The disruption of this pathway causes the complete absence of mature osteoclasts in bone biopsies (Lo Iacono et al., 2013; Pangrazio et al., 2012). Patients affected by RANKL deficiency present with severe osteopetrosis with slower progression of the disease compared to classical ARO.

Mutations in the solute carrier family 29 member 3 (*SLC29A3*) gene, encoding a lysosomal nucleoside transporter highly expressed in myeloid cells, also affect osteoclast differentiation (Campeau et al., 2012; Howaldt et al., 2019). SLC29A3 deficiency, also called dysosteosclerosis, is associated with red violet macular atrophy, platyspondyly and metaphyseal osteosclerosis.

Atypical cases of osteopetrosis have been reported in two siblings affected by severe combined immunodeficiency (SCID), who carry a mutation in the TNF receptor-associated factor 6 (*TRAF6*) gene, the most important adaptor for the RANK/RANKL signaling pathway (Weisz Hubshman et al., 2017). In addition, a heterozygous truncating mutation in the colony-stimulating factor 1 receptor (*CSF1R*) gene, which encodes the macrophage colony-stimulating factor 1 receptor (M-CSF), was reported in the consanguineous parents of two deceased siblings showing osteopetrosis and brain malformations (Monies et al., 2017).

## Consensus guidelines for the treatment of osteopetrosis

### Supportive treatments

Without treatment, ARO is lethal in 70% of cases (Gillani and Abbas, 2017). Because osteoclasts originate from hematopoietic precursors, hematopoietic stem cell transplantation (HSCT) is the therapy of choice, resulting in improved bone remodeling and reversal of pancytopenia and extramedullary hematopoiesis.

However, HSCT is effective only in cases of intrinsic osteoclast defects and is not recommended for osteopetrosis caused by absent osteoclasts due to mutation in *TNFSF11* (Even-Or and Stepensky, 2021; Schulz et al., 2015; Wu et al., 2017).

In cases of CNS involvement, such as in osteopetrosis caused by mutations in *CLCN7* and *OSTM1*, which are associated with primary neuropathy, HSCT is proven to be ineffective and contraindicated (Pangrazio et al., 2010; Schulz et al., 2015). By contrast, patients affected by intermediate osteopetrosis, comprising both severe dominant forms with an early onset and recessive ones without CNS involvement, have a better prognosis and are candidates for HSCT (Pangrazio et al., 2010). For this reason, extensive neurological evaluation by computed tomography or magnetic resonance imaging and electroencephalography are required, especially in patients carrying unknown mutations in the *CLCN7* gene (Schulz et al., 2015).

In intermediate- and late-onset forms, HSCT may pose greater risks than benefits, so the patient-specific situations must be considered. When HSCT is not indicated or adequate donors are lacking, patients are empirically treated and receive conservative care based on multi-disciplinary approaches, according to clinical manifestations (Stark and Savarirayan, 2009). These interventions may include, among others, calcium and vitamin D supplementation, corticosteroid administration, antimicrobial therapy, orthopedic surgery, neurosurgery, transfusions and pain management (Wu et al., 2017).

### HSCT: strengths and weaknesses

**Fig. 3.**
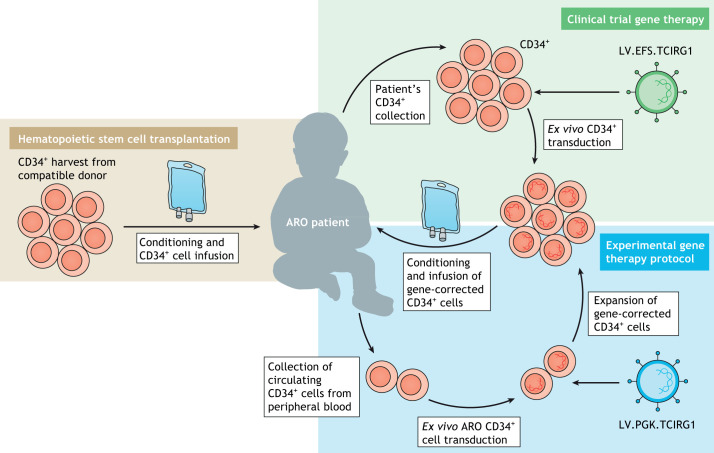
**Current therapeutic approaches for ARO and novel gene therapy strategies for *TCIRG1*-dependent osteopetrosis.** Hematopoietic stem cell (HSC) transplantation (beige box) is the current standard of care: CD34^+^ cells are collected from a compatible healthy donor and infused into the patient, who has previously received myeloablative conditioning. Alternatively, gene therapy represents an innovative approach, allowing autologous HSC transplantation without the risk of graft rejection. Currently, a Phase I clinical trial for *TCIRG1*-dependent ARO is ongoing (green box): the autosomal recessive osteopetrosis (ARO) patient's CD34^+^ cells are collected and transduced with a lentiviral vector (LV) carrying the curative gene under the control of the elongation factor 1α short (EFS) promoter*.* The transduced stem cells are later re-infused into the patient after administration of a conditioning regimen. A different approach coupling circulating ARO CD34^+^ cell expansion with LV gene correction has recently been proposed at a preclinical level by our group (blue box). In particular, circulating CD34^+^ cells are collected from the peripheral blood of ARO patients and transduced with an LV carrying the *TCIRG1* gene under the control of the phosphoglycerate kinase (PGK) promoter*.* After transduction, CD34^+^ cells are expanded *ex vivo*, exploiting the UM171-based HSC expansion protocol, which is able to increase HSC number while maintaining the cells’ stemness and engraftment capacity. The cell product is suitable for cryopreservation, thus providing the possibility to perform multiple cycles of cell infusions. Finally, the transduced and expanded HSCs are re-infused into the patient after administration of a conditioning regimen. Figure originally created using Servier Medical Art (http://smart.servier.com/), licensed under a CC-BY 3.0 license, and re-drawn according to journal style.

HSCT is the standard care for ARO patients and, although it usually improves patients' condition, it does not fully rescue the disease (Fig. 3) (Box 2). Despite full engraftment in more than 50% of transplanted ARO individuals, treated patients present progressive visual loss in the early post-transplant period (Orchard et al., 2015). Young age (less than 1 year), when disease progression and irreversible neurological damage are still limited, is a strong indication to perform HSCT. Moreover, the frequency of transplant failure increases in patients receiving HSCT after 10 months of age, particularly in a haploidentical HSCT (Box 1) setting (Schulz et al., 2015).

**Box 2. HSCT and conditioning regimens**

Hematopoietic stem cell transplantation (HSCT) is the infusion of allogeneic stem cells to reestablish hematopoietic function in patients with diseases affecting hematopoietic lineage.

To make room for donor hematopoietic stem cells (HSCs), conditioning regimens by chemotherapy are required to eliminate patients' bone marrow completely (myeloablative conditioning) or partially (reduced intensity conditioning). The alkylating agent busulfan is the most frequently used drug, because dosage can be measured and adjusted during treatment. It is usually combined with cyclophosphamide (an alkylating and immunosuppressive agent) or with the less toxic fludarabine (a highly immunosuppressive purine analog). Recently, the substitution of busulfan by treosulfan (a myeloablative and immunosuppressive alkylating agent) in reduced intensity conditioning has been explored with limited success (Schulz et al., 2015; Shadur et al., 2018).

In non-genoidentical transplants, thiotepa (an alkylating and immunosuppressive agent) and serotherapy are also administered to reduce the risk of rejection by suppressing the reaction of host T cells against the graft. Serotherapy usually consists of antithymocyte globulin (ATG), a polyclonal antibody against human thymocytes or T-cell lines, or the monoclonal antibody alemtuzumab, specific for CD52. Both types of serotherapy eliminate T cells, but they also target B and natural killer cells (Willemsen et al., 2015). Alternatively, T-cell depletion in the graft prior to haploidentical infusion reduces graft versus host disease (GvHD) but causes delayed immune recovery, making patients more susceptible to infections (Bertaina et al., 2017).

For these reasons, *in vivo* T-cell depletion has been developed and successfully employed, using post-transplant cyclophosphamide unmanipulated HSCT in osteopetrotic patients (Even-Or et al., 2021). Depletion of both donor and recipient alloreactive cells promotes engraftment and decreases risk of GvHD (Even-Or and Stepensky, 2021).

In osteopetrosis, myeloablative regimens are usually required to ensure proper donor engraftment. However, this needs to be balanced with the higher risk of conditioning toxicity in such patients. Indeed, specific complications are frequent in the autosomal recessive osteopetrosis (ARO) setting. Prophylaxis or early therapy with defibrotide is recommended to prevent venous occlusive disease of the liver, while antifungal prophylaxis may prevent post-transplant *Pneumocystic jirovecii* pneumonia. Acute pulmonary arterial hypertension often develops in ARO patients in the first months after the transplant, and combined therapy with bosentan (endothelin receptor antagonist) and sildenafil (a phosphodiesterase type 5 inhibitor) should be initiated as soon as possible.

Bone marrow transplants from human leukocyte antigen (HLA)-identical donors allow 62-88% overall survival (Driessen et al., 2003; Gerritsen et al., 1994; Orchard et al., 2015; Wynn and Schulz, 2019). In 2015, a large retrospective international study reported the outcomes of HSCT from related and unrelated donors for infantile osteopetrosis in 193 patients, with a median follow-up of 7 years (Orchard et al., 2015). This study reported overall survival of 62% at 10 years post-treatment after HLA-matched donor transplant. An improvement in survival rates has been observed, and, recently, 100% overall survival was reported in 31 patients transplanted from matched donors after reduced intensity conditioning (RIC) (Box 1) (Shadur et al., 2018).

Survival drops to 40-70% in recipients of HLA-mismatched or HLA-haploidentical donors (Schulz et al., 2015). Alternative strategies have been tested, such as T cell-depleted haploidentical HSCT (Box 1), with improved outcomes (Porta et al., 2015; Pronk et al., 2017; Schulz et al., 2002). This procedure allows an acceptable long-term donor cell engraftment and significantly limits the risk of graft versus host disease (GvHD) (Porta et al., 2015).

Another promising option is haploidentical HSCT followed by post-transplant cyclophosphamide (PT-Cy) regimens in order to eliminate alloreactive T cells and decrease the risk of GvHD (Even-Or and Stepensky, 2021; Neven et al., 2019). The use of cord blood, another standard source of cells for HSCT, is currently no longer recommended, however, because frequent primary graft failures have been recorded, resulting in poor overall survival at 3 years (Chiesa et al., 2016; Schulz et al., 2015).

HSCT treatment of ARO patients is associated with frequent adverse events, including difficulty in achieving sustained long-term donor engraftment, early transplant-related mortality, pulmonary hypertension, and veno-occlusive disease (VOD) of the liver and sepsis (Schulz et al., 2015). Coupled with the severe pre-existing clinical status of ARO patients, poor outcome and toxicities have also been associated with traditional myeloablative chemotherapy regimens, paving the way for research into new approaches for the conditioning of osteopetrotic patients to ameliorate the HSCT outcome (Even-Or and Stepensky, 2021; Wynn and Schulz, 2019). New transplant strategies such as the Baltimore protocol of T cell-replete haploidentical transplantation, involving PT-Cy administration post-transplant, may be exploited for the treatment of children older than 10 months (Fuchs, 2012; Stepensky et al., 2019), having been successful in several cases of re-transplantation after rejection or non-engraftment.

The use of fludarabine, which has a more favorable toxicity profile than cyclophosphamide, significantly improves the outcome of HSCT (Natsheh et al., 2016) and is currently recommended by European Society for Immunodeficiencies (ESID) consensus guidelines, resulting in a disease-free survival (Box 1) of 80% (Schulz et al., 2015). Of note, fludarabine-based conditioning has also been successfully employed in intermediate forms of osteopetrosis, providing a new therapeutic approach for these forms affecting adolescents or young adults (Stepensky et al., 2019).

In recent years, HSCT outcomes have been significantly improved by exploiting an RIC approach, which allows for the creation of sufficient space for HSPCs in the bone marrow compartment with minimal chemotherapy administration, thus achieving acceptable levels of donor chimerism (Box 1) and limiting the side effects of myeloablative conditioning. In particular, fludarabine is associated with improved T-cell chimerism and treosulfan with a decreased risk of VOD (Slatter et al., 2011). Accordingly, promising data have recently been obtained in osteopetrotic patients younger than 6 years old, receiving a transplant after RIC based on fludarabine, treosulfan, thiotepa and antithymocyte globulin (Shadur et al., 2018).

Despite the remarkable improvement in conditioning regimens, severe side effects like VOD, pulmonary hypertension and hypercalcemia, as well as poor donor engraftment, still hamper the transplantation outcome in ARO (Corbacioglu et al., 2006; Orchard et al., 2015; Schulz et al., 2015). Other frequent complications in osteopetrotic patients are the development of *Pneumocystic jirovecii* pneumonia (PCP), probably due to slow hematological and immunological reconstitution, although PCP can be prevented with antifungal and PCP prophylaxis; CNS complications such as malformations of cranial bones or hydrocephalous, which are reversible after HSCT; and hypocalcaemia and the risk of convulsions before the engraftment, as well as risk of hypercalcemic crisis upon successful engraftment (Schulz et al., 2015).

## Innovative therapies for ARO

In recent years, the outcome of HSCT has strongly improved as a result of new drug combinations for conditioning and RIC regimens. However, the availability of HLA-matched donors still remains an open issue, particularly for patients belonging to ethnic minorities. Because of the limited efficacy of HSCT, owing to the severity of the disease and the short time window in which patients can be treated, alternative therapeutic strategies have been developed.

### Preclinical and clinical studies

#### Lentiviral vector gene therapy

Gene therapy (GT), the therapeutic delivery of a healthy copy of the mutated gene through viral vectors, has provided a successful cure for many hematopoietic diseases in the past decades (Ferrari et al., 2021). Preclinical models of GT for *TCIRG1*-dependent osteopetrosis have been developed during these years, demonstrating the efficacy and safety of this therapeutic approach as a valid alternative for patients lacking a compatible donor.

The first attempt exploited a gammaretroviral vector, in which *TCIRG1* expression is driven by the spleen focus-forming virus (SFFV) promoter, *in vivo* in the *oc/oc* mouse model (*Tcirg1*-dependent ARO), which closely resembles the human *TCIRG1*-dependent ARO. In particular, *oc/oc* mice carry a spontaneous deletion of 1.6 kb in the 5′ end of the *Tcirg1* gene, causing the absence of ruffled border formation and defective bone resorption. Homozygous mice usually die within 3 weeks after birth due to severe bone marrow fibrosis. They are characterized by growth retardation, exhibiting radiological and histological features of osteopetrosis, such as increased skeletal density and absence of marrow cavities, clubbed feet and circling behavior, owing to cranial nerve compression and absent or delayed eruption of the incisors (Scimeca et al., 2000).

GT was able to improve bone defects and survival of 50% of the treated *oc/oc* mice, despite the low number of available mice, which is due to the extremely severe phenotype of this model (Johansson et al., 2007). Subsequently, clinical trials of GT for immunodeficiencies asserted the superiority of lentiviral vectors (LVs) over retroviral vectors in terms of efficacy and safety (Thrasher and Williams, 2017).

For these reasons, during the past decade, Richter's group exploited an LV carrying the *TCIRG1* gene under the control of the elongation factor 1α short (EFS) promoter, and confirmed the suitability of a GT approach by allowing nine out of 12 mice to survive (Löfvall et al., 2019). In this study, transduced *oc/oc* c-Kit^+^ (also known as Kit^+^) fetal liver cells were transplanted into sublethally irradiated *oc/oc* pups by temporal vein injection, 1 day after birth. Long-term (19-25 weeks)-surviving mice showed reversal of the osteopetrotic bone phenotype both *in vitro*, by osteoclast culture, and *in vivo*, through the quantification of C-terminal telopeptide of type I collagen (CTX-I) (Box 1) in the serum and histopathology analysis of the bones (Löfvall et al., 2019).

Along the same lines, these authors previously demonstrated that this clinically applicable LV can also *ex vivo* correct circulating CD34^+^ cells (Box 1) of ARO patients, which rescues the resorptive capacity of ARO patient-derived osteoclasts *in vitro* (Moscatelli et al., 2018). They also demonstrated that transduced ARO CD34^+^ cells are able to engraft in immunodeficient non-obese diabetic SCID *Il2rγ^−/−^* (NSG) mice with a rate similar to cord blood CD34^+^ cells. Additionally, human CD34^+^ cells isolated from the bone marrow and spleen of transplanted NSG mice are able to differentiate into bone-resorbing osteoclasts *in vitro* (Moscatelli et al., 2018, 2013).

Importantly, in preclinical GT studies, partial correction (in terms of mixed donor chimerism or low vector copy number) seems to be sufficient to ensure osteoclast function and could possibly lead to clinical benefits (Johansson et al., 2006; Thudium et al., 2016). These observations are in accordance with the clinical data in patients post-HSCT, which show that 5% donor chimerism (or even as low as 2% in some cases) allows sustained hematopoietic recovery and normal bones at X-ray examination (Even-Or and Stepensky, 2021; Orchard et al., 2015). Thus, GT may provide a cure for patients even if low numbers of transduced cells are transplanted and engraft in the bone marrow.

The results achieved by Richter's group paved the way for the establishment of a new phase I clinical trial (Fig. 3), which started enrolling ARO patients carrying *TCIRG1* gene mutations (NCT04525352, https://clinicaltrials.gov/ct2/show/NCT04525352).

Similar results have been obtained by our group, restoring the osteoclast activity of ARO patients *ex vivo* by using a clinically optimized LV carrying the *TCIRG1* gene under the control of the phosphoglycerate kinase (PGK) promoter. Of note, we demonstrated that transduced and expanded circulating CD34^+^ cells from ARO patients are bona fide long-term hematopoietic progenitors able to engraft in NSG mice and to repopulate multiple lineages in secondary NSG recipients (Capo et al., 2021).

#### HSC sources and collection

As already mentioned, bone marrow harvest is not feasible in ARO patients due to dense bone marrow cavity sclerosis. However, these patients present a high frequency of circulating CD34^+^ cells in their blood, which have been exploited for autologous backup before HSCT, to be reinfused in case of graft failure (Steward et al., 2005). Steward et al. (2005) reported successful granulocyte colony-stimulating factor (G-CSF)-based HSC mobilization in two patients with atypical osteopetrosis, having a poor CD34^+^ cell count in peripheral blood, to guarantee an adequate autologous backup before transplantation. However, there are no other reports of hematopoietic stem cell (HSC) mobilization in children with classical severe ARO so far. If proven safe and effective, this approach could favor the harvest of an adequate amount of CD34^+^ cells from the blood for autologous back-up transplantation (Box 1) or gene correction.

Conversely, patients affected by typical early-onset ARO present a high number of circulating CD34^+^ cells in peripheral blood. These cells are easily accessible, although exchange apheresis (Box 1) or multiple rounds of collection might be necessary to achieve an adequate cell dose. *In vitro* CD34^+^ cell expansion has been developed to increase cell number while maintaining hematopoietic cell stemness and engraftment capacity. An HSC expansion protocol, based on the small pyrimidoindole derivative UM171 (Box 1), has recently been applied in an innovative clinical trial in the context of cord blood transplantation (NCT02668315, https://clinicaltrials.gov/ct2/show/NCT02668315; Cohen et al., 2020). This protocol was successfully used for ARO circulating CD34^+^ cells after gene correction, showing long-term engraftment and multilineage reconstitution in NSG mice (Fig. 3) (Capo et al., 2021).

HSC expansion, alone or in combination with mobilization, would allow the therapeutic limitations imposed by the reduced number of cells that can be retrieved in ARO patients to be overcome, limiting the therapy burden, especially in very young and severely affected patients.

### Proof-of-concept studies

Novel therapeutic approaches are currently being studied, with the final aim to provide a safe and effective alternative treatment for ARO patients who are not eligible for current standard care (summarized in Table 1).

**Table 1.**
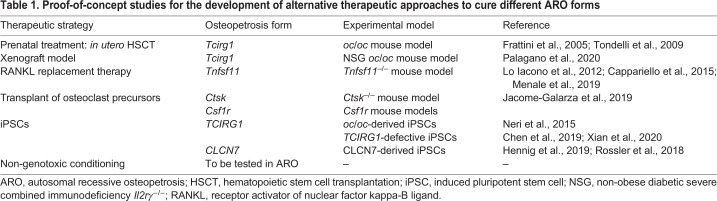
Proof-of-concept studies for the development of alternative therapeutic approaches to cure different ARO forms

#### Prenatal treatment

*In utero* transplantation of donor HSCs was tested to prevent early-onset irreversible bone defects and bone marrow fibrosis in the *oc/oc* mouse model. Pregnant females were injected *in utero* at 14.5 days post-coitum and transplant success was observed in 35% of mutant *oc/oc* mice that survived past the expected lifespan of *oc/oc* mice. Although smaller in size, they showed restored osteoclast resorption capacity, normalization of bone density and eruption of incisors, providing evidence that transplants have to be performed very early in life to avoid the development of secondary irreversible defects (Frattini et al., 2005). These data were further confirmed by a second study reporting results obtained from *in utero* injection of allogeneic fetal liver cells, and this treatment showed a similar percentage of success (Tondelli et al., 2009). Importantly, in humans, *in utero* transplantation has already been successfully performed in cases of primary immunodeficiencies (Magnani et al., 2019) and could also prevent secondary neurological damage caused by nerve compression. However, *in utero* transplantation could be applicable only to pre-term diagnosis and may be challenging in humans.

#### Novel xenograft model

Recently, Palagano et al. (2020) developed an *oc/oc* (*Tcirg1*-dependent) mouse model in the immunodeficient NSG background to study the osteoclast generation from patient cells *in vivo*, performing xenotransplantation of ARO CD34^+^ cells in an osteopetrotic setting. This murine model displayed typical NSG features, such as the complete lack of B, T and NK cells, and, in parallel, osteoclast-rich osteopetrosis with severe growth retardation, short lifespan, defective tooth eruption and extramedullary hematopoiesis. However, the short lifespan of these mice, resulting from the fast progression of the disease, did not allow a study of the corrective potential of human cells on the bone phenotype. In addition, the NSG *oc/oc* model lacks human M-CSF expression, which impedes human osteoclast differentiation, meaning that this cytokine is species specific. If implemented for the expression of human M-CSF, this model could represent a valuable tool to study the homing and engraftment of ARO patients' CD34^+^ cells, as well as the efficacy of GT, in the correct pathological context.

#### Systematic administration of deficient protein

ARO caused by osteoclast-extrinsic deficiency, such as in the case of *TNFSF11* mutations, requires a different approach, because defective cells are not of hematopoietic origin. RANKL replacement therapy has been explored in *Tnfsf11^−/−^* mice, starting early after birth. Subcutaneous delivery of recombinant soluble RANKL (sRANKL) for 1 month significantly improves bone defects and hematopoietic organ architecture, limiting nerve compression (Lo Iacono et al., 2012).

Promising results have been obtained by exploiting the use of biotechnological devices implanted subcutaneously, enabling the release of sRANKL and allowing osteoclastogenesis in *Tnfsf11^−/−^* mice. In particular, Cappariello et al. (2015) generated a bio-device harboring primary osteoblasts, cultured on 3D hydroxyapatite scaffolds carrying an immobilized metalloproteinase 14 catalytic domain, which is able to cleave RANKL into the active sRANKL. Implanted mice showed progressively improved weight and size and rescued osteoclastogenesis in tibial histological sections when multiple diffusion chambers were implanted (Cappariello et al., 2015).

Also in *Tnfsf11^−/−^* mice, Menale et al. (2019) exploited the use of a 3D culture system based on a magnesium-doped hydroxyapatite/collagen I biocompatible scaffold, which closely recapitulates bone physicochemical characteristics. The scaffold was seeded with *Tnfsf11^−/−^* mesenchymal stem cells, previously transduced with an LV overexpressing human sRANKL. Subsequently, one or two scaffolds were implanted subcutaneously in *Tnfsf11^−/−^* mice and extensive vascularization was observed after 2 months. This innovative strategy was able to drive the differentiation of TRAP (also known as ACP5)-positive osteoclasts, although osteopetrotic features were still present in bones at sacrifice (Menale et al., 2019).

The development of innovative biotechnological strategies taking advantage of a bio-device delivering sRANKL, inducing osteoclastogenesis in RANKL-deficient mice, supports the feasibility of novel approaches to treat systemic cytokine deficiencies.

#### Transplant of osteoclast precursors

A recent elegant study found that osteoclasts are long-lived syncytia that acquire new nuclei by iterative fusion of circulating blood monocytes (Jacome-Galarza et al., 2019). In particular, the authors of this study abrogated RANK or CD115 expression in the bone marrow HSC progenitors (using *Csf1r^cre^* mice) or in bone marrow HSCs, and in embryonic erythro-myeloid progenitors (EMPs) (using *Flt3^cre^* mice). They demonstrated that osteoclasts originating from EMPs are necessary for normal bone development, whereas osteoclasts originating from bone marrow HSC progenitors are essential for bone turnover in adulthood. As a consequence, transplantation of monocytic cells is able to sustain osteoclastogenesis and bone resorption in both early-onset ARO and adult-onset osteopetrotic mice, in the absence of HSC engraftment (Jacome-Galarza et al., 2019). This approach would allow repeated infusions and could be exploited as a bridge therapy before allogeneic HSCT or autologous GT.

##### Gene-corrected induced pluripotent stem cells

In the past decades, patient-derived induced pluripotent stem cells (iPSCs) have been extensively applied to investigate the pathobiology of different diseases and to test innovative treatments, thereby overcoming the limitations imposed by the availability of specimens from patients. An iPSC line generated from a *CLCN7*-defective ARO patient was used to test the integration of the Sleeping Beauty transposon (Box 1) carrying *CLCN7* cDNA (Hennig et al., 2019; Rossler, 2018).

More recently, iPSCs derived from ARO patients carrying a homozygous mutation in *TCIRG1* were generated and corrected by two different groups. Cells were transduced and genetically corrected by a retroviral (Chen et al., 2019) or lentiviral (Xian et al., 2020) vector expressing the *TCIRG1* gene and then induced to differentiate into osteoclasts, partially restoring their bone-resorptive potential. Of note, gene editing in *TCIRG1-*defective ARO has been successfully demonstrated using a murine iPSC platform derived from the *oc/oc* mouse model, in which murine iPSC clones were genetically corrected using a bacterial artificial chromosome by a homologous recombination approach (Neri et al., 2015). These approaches could provide an additional therapeutic strategy for severe forms of osteopetrosis, although safety concerns still hamper their clinical use.

##### Non-genotoxic conditioning

Successful HSCT, and autologous HSC transplantation after gene correction, requires vacating recipient HSC niches to allow donor cell engraftment and guarantee long-term hematopoietic and immune functions. As already mentioned, significant improvements in conditioning regimens have been made so far, starting with the use of RIC (Shadur et al., 2018). However, in patients with pre-existing organ toxicity or in those younger than 1 year of age, chemotherapy-based conditioning regimens are poorly tolerated and result in major morbidity and mortality. New approaches for treating these patients have recently emerged, based on the use of minimal-intensity conditioning to reduce the burden of conditioning regimens for patients affected by SCID. In particular, the use of monoclonal antibody-based conditioning specific for CD45 (also known as PTPRC) or CD117 (also known as KIT) allowed the specific depletion of the target HSPC population, avoiding a widespread toxic effect of the drug on the other cell populations. Moreover, this approach seems to be well tolerated and can achieve curative engraftment, even in patients with severe pre-existing conditions (Agarwal et al., 2019; Straathof et al., 2009).

A clinical trial exploiting an anti-CD117 antibody is currently ongoing to treat SCID patients (NCT02963064, https://clinicaltrials.gov/ct2/show/NCT02963064). In parallel, antibody drug conjugates (ADCs), originally developed for cancer therapy, are an emerging class of non-myeloablative agents. Specifically, a monoclonal antibody is bound to a drug or a toxin, which is specifically internalized into the target cells, allowing targeted cell population depletion (Abadir et al., 2019).

The efficacy of CD45.2 ADC in HSCT conditioning was demonstrated with a rat anti-mouse monoclonal antibody conjugated to saporin (SAP), a catalytic N-glycosidase ribosome-inactivating protein that halts protein synthesis (Palchaudhuri et al., 2016). In mice, CD45.2–SAP preserved normal bone marrow architecture compared to total body irradiation, which instead reduced vascular integrity and bone marrow cellularity. Mice conditioned with CD45.2–SAP rapidly recovered their peripheral myeloid cells and had a survival advantage when exposed to infections (Palchaudhuri et al., 2016). Additionally, conditioning with CD45.2–SAP resulted in significant chimerism after transplantation, even in a pathological mouse model (Castiello et al., 2021).

Another efficacious ADC is CD117–SAP immunotoxin coupled with T cell-depleting agents. It has been proven to selectively deplete more than 99% of host HSCs without causing clinically significant side effects and to favor rapid and efficient donor HSC engraftment. Importantly, blood cell counts and immune cell function are preserved and treated mice effectively respond to both viral and fungal challenges (Czechowicz et al., 2019). The CD117 ADC was also exploited successfully in MHC-mismatched allotransplantation (Li et al., 2019b) and in hemophilia A GT mice (Gao et al., 2019).

These studies demonstrate that non-genotoxic conditioning as HSCT pre-treatment could be a valid alternative to myeloablative conditioning for the treatment of a wide range of non-malignant and malignant diseases and might support GT approaches for ARO patients.

## Conclusion and future perspectives

More than ten genes have been identified so far as responsible for osteopetrosis, highlighting the relevance of molecular analysis for the clinical management of this disease. Novel techniques, including next-generation sequencing, whole-exome sequencing and gene-targeted sequencing panels, are further revealing the spectrum of molecular defects, thus impacting on the therapeutic arsenal to offer to these rare patients. *In vitro* and *in vivo* studies have provided seminal preclinical data that have allowed the development of novel cellular therapies, including GT approaches based on the use of third-generation LVs for *TCIRG1* deficiency. The severity of the disease and the increased frequency of severe side effects occurring in patients undergoing hematopoietic stem cell transplantation upon conventional conditioning are increasing safety concerns, and prompt the development of safe depleting agents able to create room for HSC engraftment in the bone marrow niche while preserving tissue integrity. The implementation of these novel approaches will allow the establishment of tailored treatment programs to guarantee a cure for all ARO patients, irrespective of the severity of their clinical conditions.

## References

[DMM048940C1] Abadir, E., Bryant, C., Larsen, S. and Clark, G. J. (2019). Targeting the niche: depleting haemopoietic stem cells with targeted therapy. *Bone Marrow. Transplant.* 54, 961-968. 10.1038/s41409-019-0445-0 30664721

[DMM048940C2] Agarwal, R., Dvorak, C. C., Prohaska, S., Long-Boyle, J., Kwon, H.-S., Brown, J. M., Weinberg, K. I., Le, A., Guttman-Klein, A., Logan, A. C. et al. (2019). Toxicity-free hematopoietic stem cell engraftment achieved with anti-CD117 monoclonal antibody conditioning. *Biol. Blood Marrow Transplant.* 25 Suppl. 3, S92. 10.1016/j.bbmt.2018.12.172

[DMM048940C3] Aker, M., Rouvinski, A., Hashavia, S., Ta-Shma, A., Shaag, A., Zenvirt, S., Israel, S., Weintraub, M., Taraboulos, A., Bar-Shavit, Z. et al. (2012). An SNX10 mutation causes malignant osteopetrosis of infancy. *J. Med. Genet.* 49, 221-226. 10.1136/jmedgenet-2011-100520 22499339

[DMM048940C4] Bertaina, A., Pitisci, A., Sinibaldi, M. and Algeri, M. (2017). T cell-depleted and T cell-replete HLA-haploidentical stem cell transplantation for non-malignant disorders. *Curr. Hematol. Malig Rep.* 12, 68-78. 10.1007/s11899-017-0364-3 28116633

[DMM048940C5] Campeau, P. M., Lu, J. T., Sule, G., Jiang, M. M., Bae, Y., Madan, S., Högler, W., Shaw, N. J., Mumm, S., Gibbs, R. A. et al. (2012). Whole-exome sequencing identifies mutations in the nucleoside transporter gene SLC29A3 in dysosteosclerosis, a form of osteopetrosis. *Hum. Mol. Genet.* 21, 4904-4909. 10.1093/hmg/dds326 22875837 PMC3607481

[DMM048940C6] Capo, V., Penna, S., Merelli, I., Barcella, M., Scala, S., Basso-Ricci, L., Draghici, E., Palagano, E., Zonari, E., Desantis, G. et al. (2021). Expanded circulating hematopoietic stem/progenitor cells as novel cell source for the treatment of TCIRG1 osteopetrosis. *Haematologica* 106, 74-86. 10.3324/haematol.2019.238261 31949009 PMC7776247

[DMM048940C7] Cappariello, A., Paone, R., Maurizi, A., Capulli, M., Rucci, N., Muraca, M. and Teti, A. (2015). Biotechnological approach for systemic delivery of membrane Receptor Activator of NF-κB Ligand (RANKL) active domain into the circulation. *Biomaterials* 46, 58-69. 10.1016/j.biomaterials.2014.12.033 25678116 PMC4337851

[DMM048940C8] Castiello, M. C., Bosticardo, M., Sacchetti, N., Calzoni, E., Fontana, E., Yamazaki, Y., Draghici, E., Corsino, C., Bortolomai, I., Sereni, L. et al. (2021). Efficacy and safety of anti-CD45-saporin as conditioning agent for RAG deficiency. *J. Allergy Clin. Immunol.* 147, 309-320.e6. 10.1016/j.jaci.2020.04.033 32387109 PMC8322962

[DMM048940C9] Chalhoub, N., Benachenhou, N., Rajapurohitam, V., Pata, M., Ferron, M., Frattini, A., Villa, A. and Vacher, J. (2003). Grey-lethal mutation induces severe malignant autosomal recessive osteopetrosis in mouse and human. *Nat. Med.* 9, 399-406. 10.1038/nm842 12627228

[DMM048940C10] Chen, W., Twaroski, K., Eide, C., Riddle, M. J., Orchard, P. J. and Tolar, J. (2019). TCIRG1 transgenic rescue of osteoclast function using induced pluripotent stem cells derived from patients with infantile malignant autosomal recessive osteopetrosis. *J. Bone Joint Surg. Am.* 101, 1939-1947. 10.2106/JBJS.19.00558 31567691

[DMM048940C11] Chiesa, R., Ruggeri, A., Paviglianiti, A., Zecca, M., Gónzalez-Vicent, M., Bordon, V., Stein, J., Lawson, S., Dupont, S., Lanino, E. et al. (2016). Outcomes after unrelated umbilical cord blood transplantation for children with osteopetrosis. *Biol. Blood Marrow Transplant.* 22, 1997-2002. 10.1016/j.bbmt.2016.07.015 27470286

[DMM048940C12] Cohen, S., Roy, J., Lachance, S., Delisle, J. S., Marinier, A., Busque, L., Roy, D. C., Barabé, F., Ahmad, I., Bambace, N. et al. (2020). Hematopoietic stem cell transplantation using single UM171-expanded cord blood: a single-arm, phase 1-2 safety and feasibility study. *Lancet Haematol.* 7, e134-e145. 10.1016/S2352-3026(19)30202-9 31704264

[DMM048940C13] Corbacioglu, S., Hönig, M., Lahr, G., Stöhr, S., Berry, G., Friedrich, W. and Schulz, A. S. (2006). Stem cell transplantation in children with infantile osteopetrosis is associated with a high incidence of VOD, which could be prevented with defibrotide. *Bone Marrow. Transplant.* 38, 547-553. 10.1038/sj.bmt.1705485 16953210

[DMM048940C14] Czechowicz, A., Palchaudhuri, R., Scheck, A., Hu, Y., Hoggatt, J., Saez, B., Pang, W. W., Mansour, M. K., Tate, T. A., Chan, Y. Y. et al. (2019). Selective hematopoietic stem cell ablation using CD117-antibody-drug-conjugates enables safe and effective transplantation with immunity preservation. *Nat. Commun.* 10, 617. 10.1038/s41467-018-08201-x 30728354 PMC6365495

[DMM048940C15] Di Zanni, E., Palagano, E., Lagostena, L., Strina, D., Rehman, A., Abinun, M., De Somer, L., Martire, B., Brown, J., Kariminejad, A. et al. (2021). Pathobiologic mechanisms of neurodegeneration in osteopetrosis derived from structural and functional analysis of 14 ClC-7 mutants. *J. Bone Miner. Res.* 36, 531-545. 10.1002/jbmr.4200 33125761

[DMM048940C16] Driessen, G. J., Gerritsen, E. J., Fischer, A., Fasth, A., Hop, W. C., Veys, P., Porta, F., Cant, A., Steward, C. G., Vossen, J. M. et al. (2003). Long-term outcome of haematopoietic stem cell transplantation in autosomal recessive osteopetrosis: an EBMT report. *Bone Marrow. Transplant.* 32, 657-663. 10.1038/sj.bmt.1704194 13130312

[DMM048940C17] Even-Or, E. and Stepensky, P. (2021). How we approach malignant infantile osteopetrosis. *Pediatr. Blood Cancer* 68, e28841. 10.1002/pbc.28841 33314591

[DMM048940C18] Even-Or, E., NaserEddin, A., Dinur Schejter, Y., Shadur, B., Zaidman, I. and Stepensky, P. (2021). Haploidentical stem cell transplantation with post-transplant cyclophosphamide for osteopetrosis and other nonmalignant diseases. *Bone Marrow. Transplant.* 56, 434-441. 10.1038/s41409-020-01040-9 32855443 PMC7450679

[DMM048940C19] Ferrari, G., Thrasher, A. J. and Aiuti, A. (2021). Gene therapy using haematopoietic stem and progenitor cells. *Nat. Rev. Genet.* 22, 216-234. 10.1038/s41576-020-00298-5 33303992

[DMM048940C20] Frattini, A., Orchard, P. J., Sobacchi, C., Giliani, S., Abinun, M., Mattsson, J. P., Keeling, D. J., Andersson, A. K., Wallbrandt, P., Zecca, L. et al. (2000). Defects in TCIRG1 subunit of the vacuolar proton pump are responsible for a subset of human autosomal recessive osteopetrosis. *Nat. Genet.* 25, 343-346. 10.1038/77131 10888887

[DMM048940C21] Frattini, A., Blair, H. C., Sacco, M. G., Cerisoli, F., Faggioli, F., Catò, E. M., Pangrazio, A., Musio, A., Rucci, F., Sobacchi, C. et al. (2005). Rescue of ATPa3-deficient murine malignant osteopetrosis by hematopoietic stem cell transplantation in utero. *Proc. Natl. Acad. Sci. USA* 102, 14629-14634. 10.1073/pnas.0507637102 16195375 PMC1253616

[DMM048940C22] Fuchs, E. J. (2012). Human leukocyte antigen-haploidentical stem cell transplantation using T-cell-replete bone marrow grafts. *Curr. Opin Hematol.* 19, 440-447. 10.1097/MOH.0b013e32835822dc 22954723

[DMM048940C23] Gao, C., Schroeder, J. A., Xue, F., Jing, W., Cai, Y., Scheck, A., Subramaniam, S., Rao, S., Weiler, H., Czechowicz, A. et al. (2019). Nongenotoxic antibody-drug conjugate conditioning enables safe and effective platelet gene therapy of hemophilia A mice. *Blood Advances* 3, 2700-2711. 10.1182/bloodadvances.2019000516 31515232 PMC6759737

[DMM048940C24] Gelb, B. D., Shi, G. P., Chapman, H. A. and Desnick, R. J. (1996). Pycnodysostosis, a lysosomal disease caused by cathepsin K deficiency. *Science* 273, 1236-1238. 10.1126/science.273.5279.1236 8703060

[DMM048940C25] Gerritsen, E. J., Vossen, J. M., Fasth, A., Friedrich, W., Morgan, G., Padmos, A., Vellodi, A., Porras, O., O'Meara, A. and Porta, F. (1994). Bone marrow transplantation for autosomal recessive osteopetrosis. A report from the Working Party on Inborn Errors of the European Bone Marrow Transplantation Group. *J. Pediatr.* 125 (6 Pt 1), 896-902. 10.1016/S0022-3476(05)82004-9 7996361

[DMM048940C26] Gillani, S. and Abbas, Z. (2017). Malignant infantile osteopetrosis. *J. Ayub Med. Coll. Abbottabad* 29, 350-352. 28718264

[DMM048940C27] Hennig, A. F., Rössler, U., Boiti, F., von der Hagen, M., Gossen, M., Kornak, U. and Stachelscheid, H. (2019). Generation of a human induced pluripotent stem cell line (BIHi002-A) from a patient with CLCN7-related infantile malignant autosomal recessive osteopetrosis. *Stem Cell Res* 35, 101367. 10.1016/j.scr.2018.101367 30763735

[DMM048940C28] Howaldt, A., Nampoothiri, S., Quell, L.-M., Ozden, A., Fischer-Zirnsak, B., Collet, C., de Vernejoul, M.-C., Doneray, H., Kayserili, H. and Kornak, U. (2019). Sclerosing bone dysplasias with hallmarks of dysosteosclerosis in four patients carrying mutations in SLC29A3 and TCIRG1. *Bone* 120, 495-503. 10.1016/j.bone.2018.12.002 30537558

[DMM048940C29] Howaldt, A., Hennig, A. F., Rolvien, T., Rössler, U., Stelzer, N., Knaus, A., Böttger, S., Zustin, J., Geißler, S., Oheim, R. et al. (2020). Adult osteosclerotic metaphyseal dysplasia with progressive osteonecrosis of the jaws and abnormal bone resorption pattern due to a LRRK1 splice site mutation. *J. Bone Miner. Res.* 35, 1322-1332. 10.1002/jbmr.3995 32119750

[DMM048940C30] Iida, A., Xing, W., Docx, M. K., Nakashima, T., Wang, Z., Kimizuka, M., Van Hul, W., Rating, D., Spranger, J., Ohashi, H. et al. (2016). Identification of biallelic LRRK1 mutations in osteosclerotic metaphyseal dysplasia and evidence for locus heterogeneity. *J. Med. Genet.* 53, 568-574. 10.1136/jmedgenet-2016-103756 27055475 PMC5769692

[DMM048940C31] Jacome-Galarza, C. E., Percin, G. I., Muller, J. T., Mass, E., Lazarov, T., Eitler, J., Rauner, M., Yadav, V. K., Crozet, L., Bohm, M. et al. (2019). Developmental origin, functional maintenance and genetic rescue of osteoclasts. *Nature* 568, 541-545. 10.1038/s41586-019-1105-7 30971820 PMC6910203

[DMM048940C32] Johansson, M., Jansson, L., Ehinger, M., Fasth, A., Karlsson, S. and Richter, J. (2006). Neonatal hematopoietic stem cell transplantation cures oc/oc mice from osteopetrosis. *Exp. Hematol.* 34, 242-249. 10.1016/j.exphem.2005.11.010 16459192

[DMM048940C33] Johansson, M. K., de Vries, T. J., Schoenmaker, T., Ehinger, M., Brun, A. C., Fasth, A., Karlsson, S., Everts, V. and Richter, J. (2007). Hematopoietic stem cell-targeted neonatal gene therapy reverses lethally progressive osteopetrosis in oc/oc mice. *Blood* 109, 5178-5185. 10.1182/blood-2006-12-061382 17332244

[DMM048940C34] Kornak, U., Kasper, D., Bösl, M. R., Kaiser, E., Schweizer, M., Schulz, A., Friedrich, W., Delling, G. and Jentsch, T. J. (2001). Loss of the ClC-7 chloride channel leads to osteopetrosis in mice and man. *Cell* 104, 205-215. 10.1016/S0092-8674(01)00206-9 11207362

[DMM048940C35] Lacey, D. L., Timms, E., Tan, H. L., Kelley, M. J., Dunstan, C. R., Burgess, T., Elliott, R., Colombero, A., Elliott, G., Scully, S. et al. (1998). Osteoprotegerin ligand is a cytokine that regulates osteoclast differentiation and activation. *Cell* 93, 165-176. 10.1016/S0092-8674(00)81569-X 9568710

[DMM048940C36] Leisle, L., Ludwig, C. F., Wagner, F. A., Jentsch, T. J. and Stauber, T. (2011). ClC-7 is a slowly voltage-gated 2Cl^−^/1H^+^-exchanger and requires Ostm1 for transport activity. *EMBO J.* 30, 2140-2152. 10.1038/emboj.2011.137 21527911 PMC3117652

[DMM048940C37] Li, L., Lv, S. S., Wang, C., Yue, H. and Zhang, Z. L. (2019a). Novel CLCN7 mutations cause autosomal dominant osteopetrosis type II and intermediate autosomal recessive osteopetrosis. *Mol. Med. Rep.* 19, 5030-5038. 10.3892/mmr.2019.10123 30942407

[DMM048940C38] Li, Z., Czechowicz, A., Scheck, A., Rossi, D. J. and Murphy, P. M. (2019b). Hematopoietic chimerism and donor-specific skin allograft tolerance after non-genotoxic CD117 antibody-drug-conjugate conditioning in MHC-mismatched allotransplantation. *Nat. Commun.* 10, 616. 10.1038/s41467-018-08202-w 30728353 PMC6365540

[DMM048940C39] Lo Iacono, N., Blair, H. C., Poliani, P. L., Marrella, V., Ficara, F., Cassani, B., Facchetti, F., Fontana, E., Guerrini, M. M., Traggiai, E. et al. (2012). Osteopetrosis rescue upon RANKL administration to Rankl(−/−) mice: a new therapy for human RANKL-dependent ARO. *J. Bone Miner. Res.* 27, 2501-2510. 10.1002/jbmr.1712 22836362

[DMM048940C40] Lo Iacono, N., Pangrazio, A., Abinun, M., Bredius, R., Zecca, M., Blair, H. C., Vezzoni, P., Villa, A. and Sobacchi, C. (2013). RANKL cytokine: from pioneer of the osteoimmunology era to cure for a rare disease. *Clin. Dev. Immunol.* 2013, 412768. 10.1155/2013/412768 23762088 PMC3671266

[DMM048940C41] Löfvall, H., Rothe, M., Schambach, A., Henriksen, K., Richter, J. and Moscatelli, I. (2019). Hematopoietic stem cell-targeted neonatal gene therapy with a clinically applicable lentiviral vector corrects osteopetrosis in *oc*/*oc* mice. *Hum. Gene. Ther.* 30, 1395-1404. 10.1089/hum.2019.047 31179768

[DMM048940C42] Lu, S.-Y., Li, M. and Lin, Y.-L. (2014). Mitf regulates osteoclastogenesis by modulating NFATc1 activity. *Exp. Cell Res.* 328, 32-43. 10.1016/j.yexcr.2014.08.018 25152440 PMC4177974

[DMM048940C43] Magnani, A., Jouannic, J.-M., Rosain, J., Gabrion, A., Touzot, F., Roudaut, C., Kracker, S., Mahlaoui, N., Toubert, A., Clave, E. et al. (2019). Successful in utero stem cell transplantation in X-linked severe combined immunodeficiency. *Blood Adv.* 3, 237-241. 10.1182/bloodadvances.2018023176 30683657 PMC6373733

[DMM048940C44] Malinin, N. L., Zhang, L., Choi, J., Ciocea, A., Razorenova, O., Ma, Y. Q., Podrez, E. A., Tosi, M., Lennon, D. P., Caplan, A. I. et al. (2009). A point mutation in KINDLIN3 ablates activation of three integrin subfamilies in humans. *Nat. Med.* 15, 313-318. 10.1038/nm.1917 19234460 PMC2857384

[DMM048940C45] Matsumoto, N., Sekiya, M., Tohyama, K., Ishiyama-Matsuura, E., Sun-Wada, G.-H., Wada, Y., Futai, M. and Nakanishi-Matsui, M. (2018). Essential role of the a3 isoform of V-ATPase in secretory lysosome trafficking via Rab7 recruitment. *Sci. Rep.* 8, 6701. 10.1038/s41598-018-24918-7 29712939 PMC5928161

[DMM048940C46] Menale, C., Campodoni, E., Palagano, E., Mantero, S., Erreni, M., Inforzato, A., Fontana, E., Schena, F., Van't Hof, R., Sandri, M. et al. (2019). Mesenchymal stromal cell-seeded biomimetic scaffolds as a factory of soluble RANKL in Rankl-deficient osteopetrosis. *Stem Cells Transl Med* 8, 22-34. 10.1002/sctm.18-0085 30184340 PMC6312453

[DMM048940C47] Mikami, T., Miake, Y., Bologna-Molina, R. and Takeda, Y. (2016). Ultrastructural analyses of alveolar bone in a patient with osteomyelitis secondary to osteopetrosis: a review of the literature. *J. Oral Maxillofac. Surg.* 74, 1584-1595. 10.1016/j.joms.2016.02.016 27000409

[DMM048940C48] Monies, D., Maddirevula, S., Kurdi, W., Alanazy, M. H., Alkhalidi, H., Al-Owain, M., Sulaiman, R. A., Faqeih, E., Goljan, E., Ibrahim, N. et al. (2017). Autozygosity reveals recessive mutations and novel mechanisms in dominant genes: implications in variant interpretation. *Genet. Med.* 19, 1144-1150. 10.1038/gim.2017.22 28383543

[DMM048940C49] Moscatelli, I., Thudium, C. S., Flores, C., Schulz, A., Askmyr, M., Gudmann, N. S., Andersen, N. M., Porras, O., Karsdal, M. A., Villa, A. et al. (2013). Lentiviral gene transfer of TCIRG1 into peripheral blood CD34(+) cells restores osteoclast function in infantile malignant osteopetrosis. *Bone* 57, 1-9. 10.1016/j.bone.2013.07.026 23907031

[DMM048940C50] Moscatelli, I., Löfvall, H., Schneider Thudium, C., Rothe, M., Montano, C., Kertész, Z., Sirin, M., Schulz, A., Schambach, A., Henriksen, K. et al. (2018). Targeting NSG mice engrafting cells with a clinically applicable lentiviral vector corrects osteoclasts in infantile malignant osteopetrosis. *Hum. Gene. Ther.* 29, 938-949. 10.1089/hum.2017.053 28726516

[DMM048940C51] Nakagawa, N., Kinosaki, M., Yamaguchi, K., Shima, N., Yasuda, H., Yano, K., Morinaga, T. and Higashio, K. (1998). RANK is the essential signaling receptor for osteoclast differentiation factor in osteoclastogenesis. *Biochem. Biophys. Res. Commun.* 253, 395-400. 10.1006/bbrc.1998.9788 9878548

[DMM048940C52] Natsheh, J., Drozdinsky, G., Simanovsky, N., Lamdan, R., Erlich, O., Gorelik, N., Or, R., Weintraub, M. and Stepensky, P. (2016). Improved outcomes of hematopoietic stem cell transplantation in patients with infantile malignant osteopetrosis using fludarabine-based conditioning. *Pediatr. Blood Cancer* 63, 535-540. 10.1002/pbc.25801 26485304

[DMM048940C53] Neri, T., Muggeo, S., Paulis, M., Caldana, M. E., Crisafulli, L., Strina, D., Focarelli, M. L., Faggioli, F., Recordati, C., Scaramuzza, S. et al. (2015). Targeted gene correction in osteopetrotic-induced pluripotent stem cells for the generation of functional osteoclasts. *Stem Cell Rep.* 5, 558-568. 10.1016/j.stemcr.2015.08.005 PMC4624934 26344905

[DMM048940C54] Neven, B., Diana, J. S., Castelle, M., Magnani, A., Rosain, J., Touzot, F., Moreira, B., Fremond, M. L., Briand, C., Bendavid, M. et al. (2019). Haploidentical hematopoietic stem cell transplantation with post-transplant cyclophosphamide for primary immunodeficiencies and inherited disorders in children. *Biol. Blood Marrow Transplant.* 25, 1363-1373. 10.1016/j.bbmt.2019.03.009 30876929

[DMM048940C55] Novarino, G., Weinert, S., Rickheit, G. and Jentsch, T. J. (2010). Endosomal chloride-proton exchange rather than chloride conductance is crucial for renal endocytosis. *Science* 328, 1398. 10.1126/science.1188070 20430975

[DMM048940C56] Orchard, P. J., Fasth, A. L., Le Rademacher, J., He, W., Boelens, J. J., Horwitz, E. M., Al-Seraihy, A., Ayas, M., Bonfim, C. M., Boulad, F. et al. (2015). Hematopoietic stem cell transplantation for infantile osteopetrosis. *Blood* 126, 270-276. 10.1182/blood-2015-01-625541 26012570 PMC4497967

[DMM048940C57] Palagano, E., Blair, H. C., Pangrazio, A., Tourkova, I., Strina, D., Angius, A., Cuccuru, G., Oppo, M., Uva, P., Van Hul, W. et al. (2015). Buried in the middle but guilty: intronic mutations in the TCIRG1 gene cause human autosomal recessive osteopetrosis. *J. Bone Miner. Res.* 30, 1814-1821. 10.1002/jbmr.2517 25829125

[DMM048940C58] Palagano, E., Menale, C., Sobacchi, C. and Villa, A. (2018). Genetics of osteopetrosis. *Curr. Osteoporos Rep.* 16, 13-25. 10.1007/s11914-018-0415-2 29335834

[DMM048940C59] Palagano, E., Muggeo, S., Crisafulli, L., Tourkova, I. L., Strina, D., Mantero, S., Fontana, E., Locatelli, S. L., Monari, M., Morenghi, E. et al. (2020). Generation of an immunodeficient mouse model of tcirg1-deficient autosomal recessive osteopetrosis. *Bone Rep.* 12, 100242. 10.1016/j.bonr.2020.100242 31938717 PMC6953598

[DMM048940C60] Palchaudhuri, R., Saez, B., Hoggatt, J., Schajnovitz, A., Sykes, D. B., Tate, T. A., Czechowicz, A., Kfoury, Y., Ruchika, F., Rossi, D. J. et al. (2016). Non-genotoxic conditioning for hematopoietic stem cell transplantation using a hematopoietic-cell-specific internalizing immunotoxin. *Nat. Biotechnol.* 34, 738-745. 10.1038/nbt.3584 27272386 PMC5179034

[DMM048940C61] Pangrazio, A., Pusch, M., Caldana, E., Frattini, A., Lanino, E., Tamhankar, P. M., Phadke, S., Lopez, A. G., Orchard, P., Mihci, E. et al. (2010). Molecular and clinical heterogeneity in CLCN7-dependent osteopetrosis: report of 20 novel mutations. *Hum. Mutat.* 31, E1071-E1080. 10.1002/humu.21167 19953639

[DMM048940C62] Pangrazio, A., Cassani, B., Guerrini, M. M., Crockett, J. C., Marrella, V., Zammataro, L., Strina, D., Schulz, A., Schlack, C., Kornak, U. et al. (2012). RANK-dependent autosomal recessive osteopetrosis: characterization of five new cases with novel mutations. *J. Bone Miner. Res.* 27, 342-351. 10.1002/jbmr.559 22271396 PMC3306792

[DMM048940C63] Pangrazio, A., Fasth, A., Sbardellati, A., Orchard, P. J., Kasow, K. A., Raza, J., Albayrak, C., Albayrak, D., Vanakker, O. M., De Moerloose, B. et al. (2013). SNX10 mutations define a subgroup of human autosomal recessive osteopetrosis with variable clinical severity. *J. Bone Miner. Res.* 28, 1041-1049. 10.1002/jbmr.1849 23280965

[DMM048940C64] Penna, S., Capo, V., Palagano, E., Sobacchi, C. and Villa, A. (2019). One disease, many genes: implications for the treatment of osteopetroses. *Front. Endocrinol.* 10, 85. 10.3389/fendo.2019.00085 PMC6389615 30837952

[DMM048940C65] Porta, F., Cavagnini, S., Imberti, L., Sottini, A., Bolda, F., Beghin, A., Caruso, A. and Lanfranchi, A. (2015). Partial depletion of TCR alpha/beta(+)/ CD19(+) cells in matched unrelated transplantation of three patients with osteopetrosis. *Bone Marrow. Transplant.* 50, 1583-1585. 10.1038/bmt.2015.201 26367231

[DMM048940C66] Pronk, C. J., Turkiewicz, D., Vult von Steyern, K., Ehinger, M., Dykes, J. and Toporski, J. (2017). Transplantation of haploidentical TcRaß-depleted hematopoietic cells allows for optimal timing and sustained correction of the metabolic defect in children with infantile osteopetrosis. *J. Bone Miner. Res.* 32, 82-85. 10.1002/jbmr.2921 27447118

[DMM048940C67] Rossler, U. (2018). Osteoclasts differentiated from iPS cells as a test system for gene therapeutic approaches for autosomal recessive osteopetrosis (P187). In *Abstracts of the ECTS Congress*, 2018 ed. Vol. 102 (eds. H. S. Floriane Hennig, G. Manfred, K. Zsuzsanna Izsva and K. Uwe), pp. S1-S159. Calcif Tissue Int. 10.1007/s00223-018-0418-0.

[DMM048940C68] Schinke, T., Schilling, A. F., Baranowsky, A., Seitz, S., Marshall, R. P., Linn, T., Blaeker, M., Huebner, A. K., Schulz, A., Simon, R. et al. (2009). Impaired gastric acidification negatively affects calcium homeostasis and bone mass. *Nat. Med.* 15, 674-681. 10.1038/nm.1963 19448635

[DMM048940C69] Schmidt, S., Nakchbandi, I., Ruppert, R., Kawelke, N., Hess, M. W., Pfaller, K., Jurdic, P., Fässler, R. and Moser, M. (2011). Kindlin-3-mediated signaling from multiple integrin classes is required for osteoclast-mediated bone resorption. *J. Cell Biol.* 192, 883-897. 10.1083/jcb.201007141 21357746 PMC3051823

[DMM048940C70] Schulz, A. S., Classen, C. F., Mihatsch, W. A., Sigl-Kraetzig, M., Wiesneth, M., Debatin, K. M., Friedrich, W. and Müller, S. M. (2002). HLA-haploidentical blood progenitor cell transplantation in osteopetrosis. *Blood* 99, 3458-3460. 10.1182/blood.V99.9.3458 11964318

[DMM048940C71] Schulz, A. S., Moshous, D., Steward, C. G., Villa, A. and Sobacchi, C. (2015). Osteopetrosis. Consensus guidelines for diagnosis, therapy and follow-up. ESID and the EBMT WP Inborn Errors.

[DMM048940C72] Scimeca, J. C., Franchi, A., Trojani, C., Parrinello, H., Grosgeorge, J., Robert, C., Jaillon, O., Poirier, C., Gaudray, P. and Carle, G. F. (2000). The gene encoding the mouse homologue of the human osteoclast-specific 116-kDa V-ATPase subunit bears a deletion in osteosclerotic (oc/oc) mutants. *Bone* 26, 207-213. 10.1016/s8756-3282(99)00278-1 10709991

[DMM048940C73] Shadur, B., Zaidman, I., NaserEddin, A., Lokshin, E., Hussein, F., Oron, H. C., Avni, B., Grisariu, S. and Stepensky, P. (2018). Successful hematopoietic stem cell transplantation for osteopetrosis using reduced intensity conditioning. *Pediatr. Blood Cancer* 65, e27010. 10.1002/pbc.27010 29469225

[DMM048940C74] Slatter, M. A., Rao, K., Amrolia, P., Flood, T., Abinun, M., Hambleton, S., Nademi, Z., Goulden, N., Davies, G., Qasim, W. et al. (2011). Treosulfan-based conditioning regimens for hematopoietic stem cell transplantation in children with primary immunodeficiency: United Kingdom experience. *Blood* 117, 4367-4375. 10.1182/blood-2010-10-312082 21325599

[DMM048940C75] Sly, W. S., Hewett-Emmett, D., Whyte, M. P., Yu, Y. S. and Tashian, R. E. (1983). Carbonic anhydrase II deficiency identified as the primary defect in the autosomal recessive syndrome of osteopetrosis with renal tubular acidosis and cerebral calcification. *Proc. Natl. Acad. Sci. USA* 80, 2752-2756. 10.1073/pnas.80.9.2752 6405388 PMC393906

[DMM048940C76] Sobacchi, C., Villa, A., Schulz, A. and Kornak, U. (2007). CLCN7-Related Osteopetrosis. In *GeneReviews^®^* (eds. M. P. Adam, H. H. Ardinger, R. A. Pagon, S. E. Wallace, L. J. H. Bean, K. Stephens and A. Amemiya). Seattle, WA: University of Washington, Seattle.

[DMM048940C77] Sobacchi, C., Schulz, A., Coxon, F. P., Villa, A. and Helfrich, M. H. (2013). Osteopetrosis: genetics, treatment and new insights into osteoclast function. *Nat. Rev. Endocrinol.* 9, 522-536. 10.1038/nrendo.2013.137 23877423

[DMM048940C78] Stark, Z. and Savarirayan, R. (2009). Osteopetrosis. *Orphanet J. Rare Dis.* 4, 5. 10.1186/1750-1172-4-5 19232111 PMC2654865

[DMM048940C79] Stepensky, P., Grisariu, S., Avni, B., Zaidman, I., Shadur, B., Elpeleg, O., Sirin, M., Hoenig, M., Schuetz, C., Furlan, I. et al. (2019). Stem cell transplantation for osteopetrosis in patients beyond the age of 5 years. *Blood Adv.* 3, 862-868. 10.1182/bloodadvances.2018025890 30885997 PMC6436016

[DMM048940C80] Steward, C. G., Blair, A., Moppett, J., Clarke, E., Virgo, P., Lankester, A., Burger, S. R., Sauer, M. G., Flanagan, A. M., Pamphilon, D. H. et al. (2005). High peripheral blood progenitor cell counts enable autologous backup before stem cell transplantation for malignant infantile osteopetrosis. *Biol. Blood Marrow Transplant.* 11, 115-121. 10.1016/j.bbmt.2004.11.001 15682072

[DMM048940C81] Straathof, K. C., Rao, K., Eyrich, M., Hale, G., Bird, P., Berrie, E., Brown, L., Adams, S., Schlegel, P. G., Goulden, N. et al. (2009). Haemopoietic stem-cell transplantation with antibody-based minimal-intensity conditioning: a phase 1/2 study. *Lancet* 374, 912-920. 10.1016/S0140-6736(09)60945-4 19729196

[DMM048940C82] Thrasher, A. J. and Williams, D. A. (2017). Evolving gene therapy in primary immunodeficiency. *Mol. Ther.* 25, 1132-1141. 10.1016/j.ymthe.2017.03.018 28366768 PMC5417846

[DMM048940C83] Thudium, C. S., Moscatelli, I., Löfvall, H., Kertész, Z., Montano, C., Bjurström, C. F., Karsdal, M. A., Schulz, A., Richter, J. and Henriksen, K. (2016). Regulation and function of lentiviral vector-mediated TCIRG1 expression in osteoclasts from patients with infantile malignant osteopetrosis: implications for gene therapy. *Calcif. Tissue Int.* 99, 638-648. 10.1007/s00223-016-0187-6 27541021

[DMM048940C84] Tondelli, B., Blair, H. C., Guerrini, M., Patrene, K. D., Cassani, B., Vezzoni, P. and Lucchini, F. (2009). Fetal liver cells transplanted in utero rescue the osteopetrotic phenotype in the oc/oc mouse. *Am. J. Pathol.* 174, 727-735. 10.2353/ajpath.2009.080688 19218349 PMC2665735

[DMM048940C85] Van Wesenbeeck, L., Odgren, P. R., Coxon, F. P., Frattini, A., Moens, P., Perdu, B., MacKay, C. A., Van Hul, E., Timmermans, J. P., Vanhoenacker, F. et al. (2007). Involvement of PLEKHM1 in osteoclastic vesicular transport and osteopetrosis in incisors absent rats and humans. *J. Clin. Invest.* 117, 919-930. 10.1172/JCI30328 17404618 PMC1838941

[DMM048940C86] Weisz Hubshman, M., Basel-Vanagaite, L., Krauss, A., Konen, O., Levy, Y., Garty, B. Z., Smirin-Yosef, P., Maya, I., Lagovsky, I., Taub, E. et al. (2017). Homozygous deletion of RAG1, RAG2 and 5′ region TRAF6 causes severe immune suppression and atypical osteopetrosis. *Clin. Genet.* 91, 902-907. 10.1111/cge.12916 27808398

[DMM048940C87] Willemsen, L., Jol-van der Zijde, C. M., Admiraal, R., Putter, H., Jansen-Hoogendijk, A. M., Ostaijen-Ten Dam, M. M., Wijnen, J. T., van Kesteren, C., Waaijer, J. L., Lankester, A. C. et al. (2015). Impact of serotherapy on immune reconstitution and survival outcomes after stem cell transplantations in children: thymoglobulin versus alemtuzumab. *Biol. Blood Marrow Transplant.* 21, 473-482. 10.1016/j.bbmt.2014.11.674 25485863

[DMM048940C88] Wu, C. C., Econs, M. J., DiMeglio, L. A., Insogna, K. L., Levine, M. A., Orchard, P. J., Miller, W. P., Petryk, A., Rush, E. T., Shoback, D. M. et al. (2017). Diagnosis and management of osteopetrosis: consensus guidelines from the osteopetrosis working group. *J. Clin. Endocrinol. Metab.* 102, 3111-3123. 10.1210/jc.2017-01127 28655174

[DMM048940C89] Wynn, R. and Schulz, A. (2019). Inborn errors of metabolism and osteopetrosis. In *The EBMT Handbook: Hematopoietic Stem Cell Transplantation and Cellular Therapies* (ed. E. Carreras, C. Dufour, M. Mohty and N. Kröger), pp. 671-676. Cham, CH: Springer. 32091673

[DMM048940C90] Xian, X., Moraghebi, R., Löfvall, H., Fasth, A., Henriksen, K., Richter, J., Woods, N.-B. and Moscatelli, I. (2020). Generation of gene-corrected functional osteoclasts from osteopetrotic induced pluripotent stem cells. *Stem Cell Res. Ther.* 11, 179. 10.1186/s13287-020-01701-y 32414402 PMC7227215

[DMM048940C91] Zirngibl, R. A., Wang, A., Yao, Y., Manolson, M. F., Krueger, J., Dupuis, L., Mendoza-Londono, R. and Voronov, I. (2019). Novel c.G630A TCIRG1 mutation causes aberrant splicing resulting in an unusually mild form of autosomal recessive osteopetrosis. *J. Cell. Biochem.* 120, 17180-17193. 10.1002/jcb.28979 31111556

